# Habitat suitability for the invasion of *Bombus terrestris* in East Asian countries: A case study of spatial overlap with local Chinese bumblebees

**DOI:** 10.1038/s41598-018-29414-6

**Published:** 2018-07-23

**Authors:** Muhammad Naeem, Xiaolong Yuan, Jiaxing Huang, Jiandong An

**Affiliations:** Key Laboratory for Insect-Pollinator Biology of the Ministry of Agriculture, Institute of Apicultural Research, Chinese Academy of Agricultural Sciences, Beijing, 100093 China

## Abstract

Invasive species such as *Bombus terrestris* represent a severe threat that can result in the decline of local biodiversity. We examined the habitat suitability for *B*. *terrestris* invasion in East Asian countries and the risk of habitat overlap with 24 bumblebee species from different groups in China. All East Asian countries were predicted to be susceptible to invasion by *B*. *terrestris*, with the highest habitat suitability occurring in China followed by Japan, North Korea, South Korea and Mongolia. Within China, which is a global biodiversity hotspot for bumblebees, three different regions, north-west, central to south-east and north-east, were predicted as being highly suitable for invasion. In China, the group of species closely related to *B*. *terrestris* showed higher sensitivity (89%) to habitat overlap with *B*. *terrestris* than did the group of non-closely related species (73%). The risk of overlap for the three major regional bumblebees within China decreased in the order southern region, northern region and Tibetan Plateau region. Due to the sensitivity of habitat suitability and overlap, the use of the introduced European bumblebee *B*. *terrestris* for pollination services should be discouraged in regions with overlapping habitats in China, and management strategies should be implemented to conserve the vulnerable bumblebees in all East Asian countries.

## Introduction

Local biodiversity faces a serious threat from the introduction of alien invasive species, which can result in extinctions or population declines^1–5^. The trade of invasive species facilitates the establishment of non-native species in new areas^6^. In many cases, the intentional introduction of non-native species represents an important benefit to economies^7^. However, the trade of non-native species, *e*.*g*., *Bombus terrestris*, produces conflicts between the associated benefits and problems^1,3,8–10^.

In approximately 1900, a European bumblebee, *Bombus terrestris* (Hymenoptera: Apidae), was first introduced in New Zealand for pollination services. Currently, it is an invasive species and has spread well beyond its native range^10–13^. Since the 1980s, with the development of greenhouse agriculture, millions of colonies of bumblebees (*B*. *terrestris*) have been produced per year as managed pollinators to meet the demands of pollination services worldwide. Trade in bumblebee colonies has provoked concerns over the invasion of *B*. *terrestris* in regards to competitive displacement and the potential for horizontal disease transmission to local bumblebees^5,14–21^ via accidental escape of queens from greenhouses that establish colonies in areas where this bumblebee is non-native^10^. Recently, North America, Japan, Chile, and Argentina have faced challenges due to the establishment of *B*. *terrestris* and population declines of local bumblebee species^3,16,22^.

Among countries worldwide, China has the richest bumblebee diversity; 125 species are found in China, representing 50% of the total number of bumblebee species worldwide. Bumblebee pollinators play significant roles in agricultural and natural ecosystems^23,24^. Although *B*. *terrestris* is now part of the Chinese bumblebee biodiversity, it originated in Europe and has been expanding its range from Europe to Central Asia, including into northwest Xinjiang, the westernmost region of China bordering Kazakhstan^25,26^. Four decades ago, bumblebees collected in Xinjiang were considered *B*. *lucorum*, but recent DNA barcoding has confirmed that the *B*. *lucorum* complex in this region now includes *B*. *terrestris*^27,28^. The continued expansion of this species towards eastern China has been restricted by the natural barriers of the Takla Makan Desert, the Badain Jaran Desert and the high-altitude Tibetan Plateau between the western and eastern parts of China. In the past 10 years, commercial *B*. *terrestris* colonies from Europe have increasingly been introduced into China and some East Asian countries to meet the demands of crop pollination in greenhouses. This practice has raised the question of whether there are risks to the habitats of native bumblebees from this introduced species. They might pose a serious threat to native bumblebees considering the high likelihood that native bumblebees they will interact with *B*. *terrestris* around flowers^29^. Habitat or range contact (overlap) can enhance interspecific competition between species^30^, and *B*. *terrestris* introduction has already disturbed the natural mating of closely related local bumblebee species in Japan^31^. An analysis of habitat overlap risk is necessary to identify the areas that may have favourable conditions for interspecific competition between *B*. *terrestris* and vulnerable local bumblebee species. Such an analysis can be performed through spatial distribution modelling (SDM)^32^ and GIS (geographical information system) approaches. SDM techniques are considered good predictive tools and have been used previously to model the distribution of invasive or unknown species^33,34^. These approaches are among the most important for developing conservation strategies for declining bumblebee species^19,35^. These techniques combine climatic data and species occurrence records to identify the most suitable environmental conditions for population maintenance or possible species overlap. If the niches of different bumblebee species overlap, interspecific competition for food resources and habitats between invasive and local bumblebee species may occur. As overall biases and uncertainty are inherent to different prediction algorithm models, these models predict distributions differently^36–38^. Therefore, in the present study, five modelling approaches were used to identify the areas most suitable for *B*. *terrestris*; the results may guide the development of effective importation regulations for this species within East Asian countries, including China. This study attempts to answer the following questions: (1) Are all East Asian countries susceptible to *B*. *terrestris* invasion? (2) What regions of China offer potential habitats facilitating *B*. *terrestris* development and establishment? (3) Are some groups of local bumblebee species more sensitive than others to habitat overlap with *B*. *terrestris* within China?

## Results

### Modelling accuracy

The area-under-the-curve (AUC) values showed average to excellent model performances (0.77~0.98). However, variation in model performance from poor to excellent was observed based on the true skilled statistics (TSS) values (0~0.98). The ranges of TSS values were 0~0.86, 0.27~0.97, 0.35~0.95, 0.40~0.93 and 0~0.98 for Envelope Score, Environmental Distance, Genetic Algorithm for Rule Set Prediction (GARP), MaxEnt and Support Vector Machine (SVM), respectively (see Supplementary Table S1). The highest modelling accuracy was found with MaxEnt for almost all 25 species including *B*. *terrestris*, and the models with the next highest levels of accuracy were GARP and Environmental Distance, Envelope Score and SVM for 24, 19, 19, 7 and 1 species, respectively, under the receiver operating characteristics curves (ROC) threshold (see Supplementary Fig. S1). Different models (with TSS >0.5) were selected for each species to calculate the final “summed” distribution maps; however, for six species, *B*. *ignitus*, *B*. *longipennis*, *B*. *lucorum*, *B*. *patagiatus*, *B*. *picipes* and *B*. *pyrosoma*, only one modelling algorithm had a TSS >0.5 (see Supplementary Table S1).

### Potential distributions of bumblebee species

**Figure 1:**
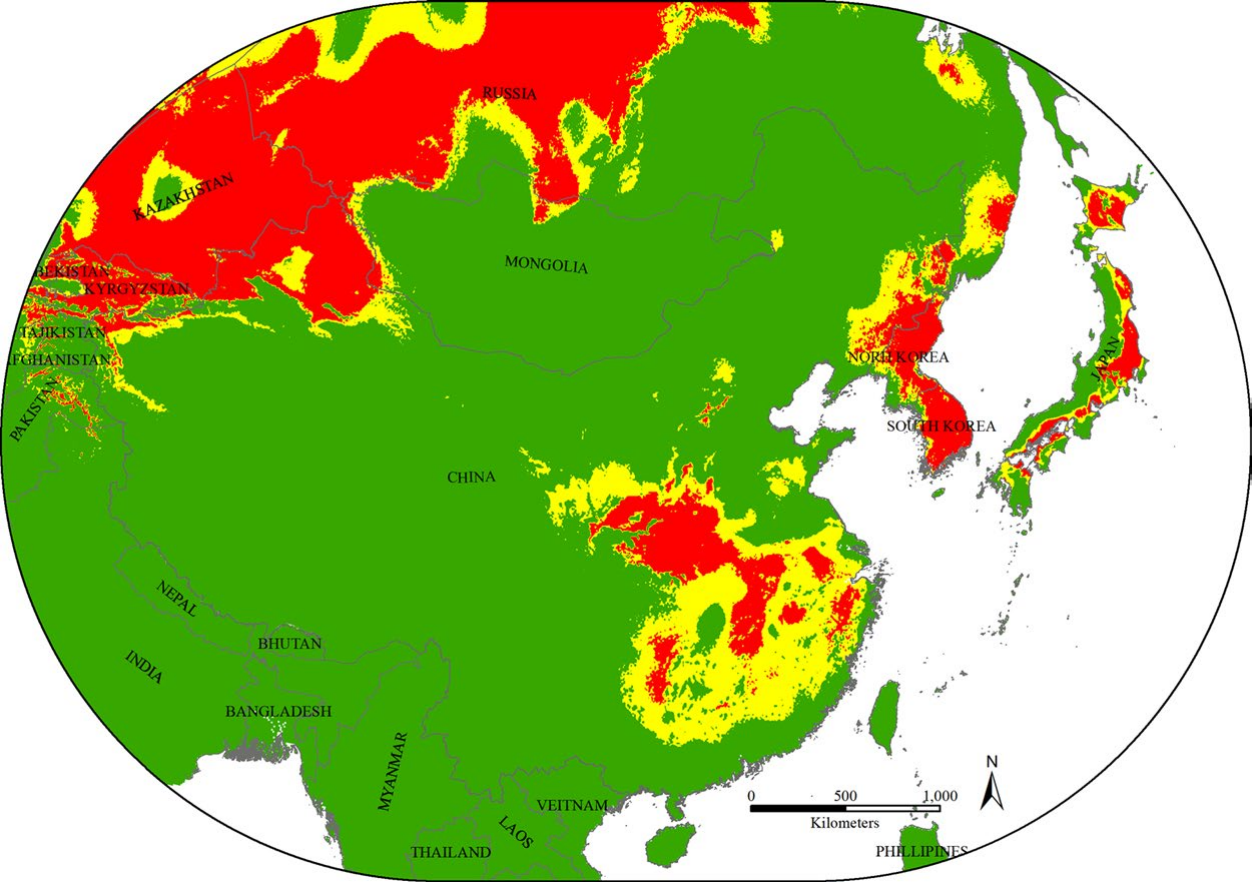
Potential distribution of *Bombus terrestris* in East Asian countries. Red represents highly suitable habitat, yellow represents suitable habitat, and green represents unsuitable habitat for *B. terrestris*. The map was created with ArcGIS v 10.0 (www.arcgis.com).

**Figure 2:**
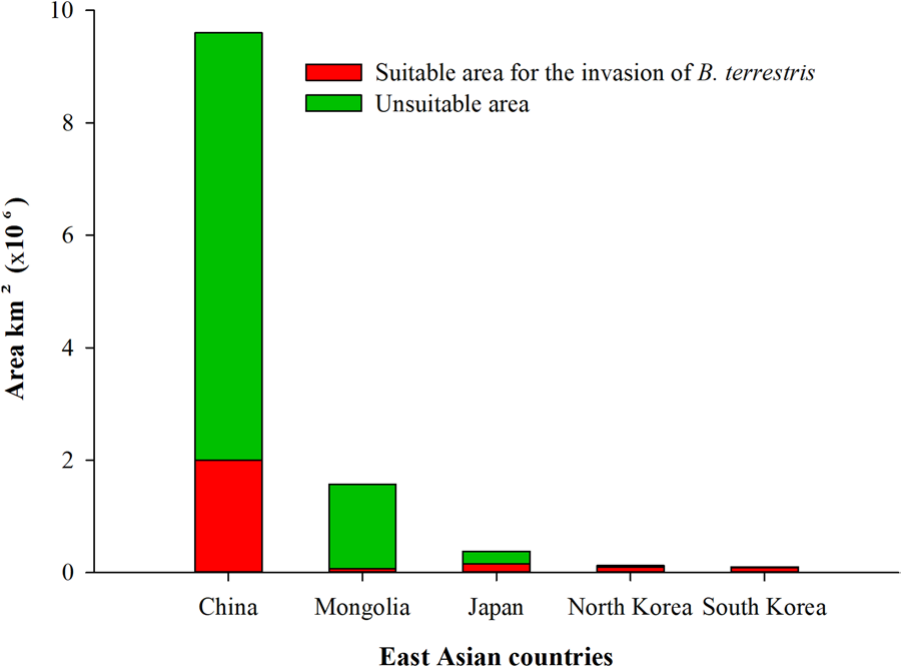
Suitable habitat areas (km^2^) for the invasion of *Bombus terrestris* in all East Asian countries.

**Figure 3:**
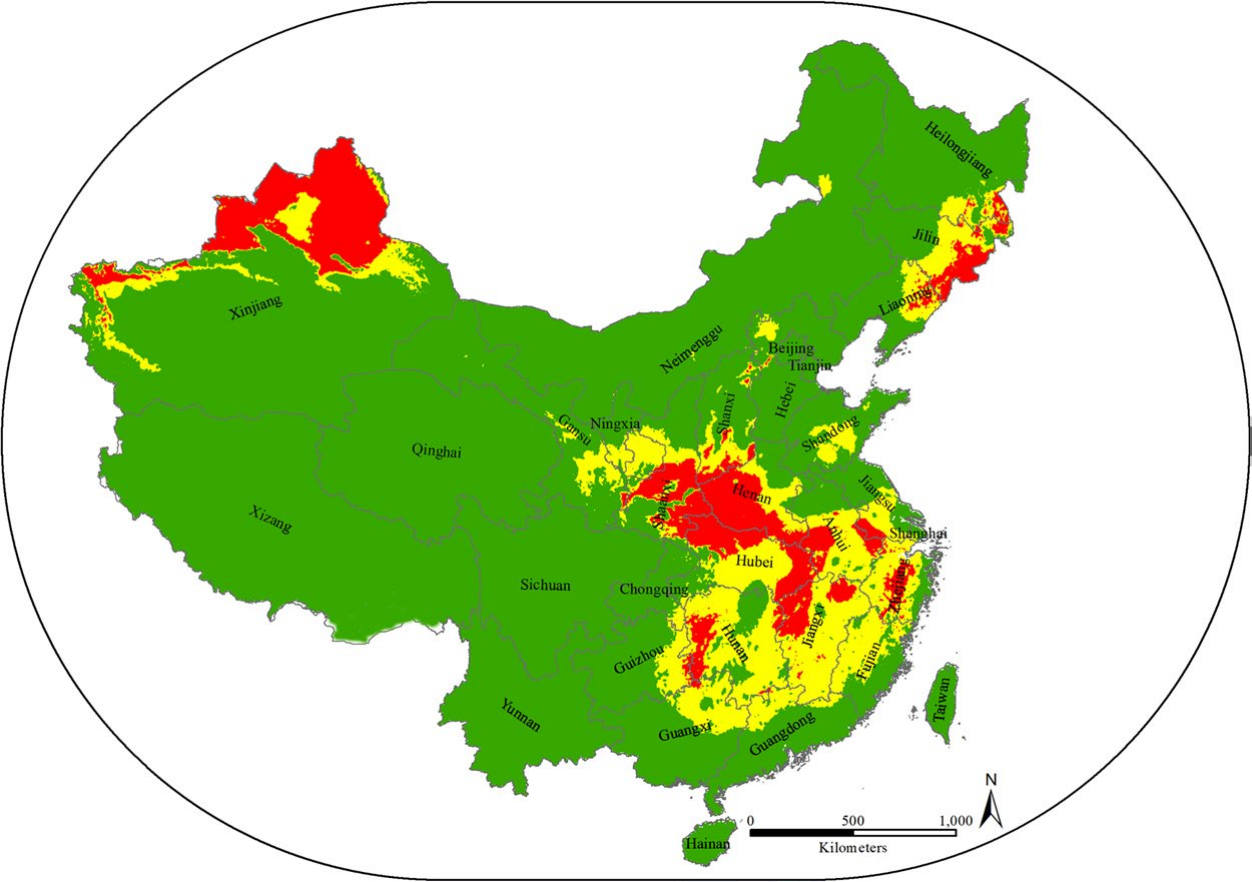
Map of the Chinese mainland showing the predicted distribution of *Bombus terrestris* with the names of the provinces in China. Red represents the highly suitable habitat, yellow represents suitable habitat, and green represents unsuitable habitat for *B. terrestris*. The map was created with ArcGIS v 10.0 (www.arcgis.com).

Predicted “suitable” and “highly suitable” areas for *B*. *terrestris* were detected in all East Asian countries, with the highest risk of invasion occurring in China, followed by Japan, North Korea, South Korea and Mongolia (Figs 1 & 2). However, within China, three different regions, north-west, central to south-east and north-east, were determined to be at risk; these areas cover an area of 2,001,333 km^2^, representing 21% of the total study area. *B*. *terrestris* expanded originally from Europe to the north-west part of China, so the central to south-east and north-east parts of China are new potential habitats for *B*. *terrestris* to become established and develop. These three zones cover 24 provinces considered to be under the threat of invasion from *B*. *terrestris*: Xinjiang, Gansu, Ningxia, Shaanxi, Shanxi, Hebei, Henan, Shandong, Chongqing, Hubei, Hunan, Guizhou, Guangxi, Guangdong, Fujian, Jiangxi, Anhui, Jiangsu, Shanghai, Zhejiang, Neimenggu, Liaoning, Jilin and Heilongjiang (Fig. 3).

The modelling results revealed the potential distributions of all local bumblebee species across the different regions of China. The potential “suitable” and “highly suitable” areas for these 25 species ranged from 665,430 to 5,707,845 km^2^. The species with the largest distribution area, representing 59% of the total study area of China, was *B*. *sibiricus*, and *B*. *longipennis* had the smallest predicted distribution area, representing 7% of the total country area (Table 1). The optimum cut-off values for all 25 species ranged from 0.44~0.91, as shown in Table 1^39^.

**Table 1:** Bumblebee species, their distribution, abundance, and occurrence records used for developing the models; Youden Index values; area under the curve (training and testing); potential suitable areas; overlapping areas; and vulnerability status within China.

Species	Subgenus	Regional distribution within China	Mean altitude (m)	Number of records before rarefying	Number of records after rarefying	Youden Index value	Average AUC of training	Average AUC of test	Potential suitable habitat area (km^2^)	Area of overlap with *B*. *terrestris* (km^2^)
*B*. *bicoloratus*	*Megabombus*	Southern	1004	469	80	0.66	0.81	0.79	2287808	831105
*B*. braccatus^*+^	*Orientalibombus*	Southern	470	80	13	0.7	0.86	0.82	1606304	1063179
*B*. *breviceps*	*Alpigenobombus*	Southern	1350	1078	156	0.72	0.9	0.89	2652191	1063179
*B*. convexus^*^	*Mendacibombus*	Tibetan Plateau	3411	198	30	0.74	0.84	0.85	1503113	0
*B*. *cryptarum*^+^	*Bombus*	Northern	1464	72	25	0.79	0.9	0.86	3163249	776801
*B*. *friseanus*	*Melanobombus*	Tibetan Plateau	2874	3205	152	0.81	0.94	0.92	2705394	0
*B*. *hypocrita*^+^	*Bombus*	Northern	428	90	17	0.91	0.99	0.98	1662583	233264
*B*. *ignitus*	*Bombus*	Northern	1029	2024	193	0.33	0.81	0.77	1124966	357782
*B*. *ladakhensis*	*Melanobombus*	Tibetan Plateau	4076	425	73	0.6	0.83	0.79	2358707	0
*B*. *lantschouensis*	*Bombus*	Northern	1803	2716	171	0.61	0.86	0.81	1074287	132155
*B*. *longipennis*	*Bombus*	Tibetan Plateau	2558	533	64	0.4	0.78	0.77	665430	0
*B*. longipes^*^	*Melanobombus*	Northern	1403	549	73	0.66	0.8	0.79	1636995	421193
*B*. *lucorum*^+^	*Bombus*	Northern	2042	19	6	0.44	0.87	0.82	1516187	490283
*B*. minshaenensis^*^	*Bombus*	Tibetan Plateau	3592	289	34	0.7	0.85	0.83	1633080	114425
*B*. *muscorum*^+^	*Thoracobombus*	Northern	1149	50	14	0.83	0.87	0.79	3760440	763978
*B*. *patagiatus*	*Bombus*	Northern	1356	2484	176	0.46	0.82	0.8	1760054	386371
*B*. picipes^*^	*Pyrobombus*	Southern	1594	169	111	0.49	0.8	0.79	1456822	389307
*B*. pyrosoma^*^	*Melanobombus*	Northern	1636	4927	270	0.33	0.81	0.78	844057	237961
*B*. *rufofasciatus*	*Melanobombus*	Tibetan Plateau	3709	1328	133	0.71	0.87	0.86	2834242	109520
*B*. *sibiricus*	*Sibiricobombus*	Northern	1828	1540	81	0.67	0.89	0.83	5707845	721585
*B*. *sporadicus*	*Bombus*	Northern	1393	107	14	0.89	0.89	0.85	1473750	223735
*B*. supremus^*+^	*Megabombus*	Tibetan Plateau	4200	88	23	0.72	0.87	0.83	1923915	0
*B*. *terrestris*^+^	*Bombus*	Northern	1307	240	15	0.73	0.91	0.86	2585636	—
*B*. trifasciatus^*^	*Megabombus*	Southern	1010	3484	246	0.69	0.89	0.86	2576406	1160507
*B*. *ussurensis*	*Megabombus*	Northern	402	333	50	0.7	0.85	0.88	2435097	286585

^*^Endemic species to China, ^+^Rare in abundance in China.

Six of seven native Tibetan bumblebee species, *B*. *convexus*, *B*. *minshaenensis*, *B*. *rufofasciatus*, *B*. *supremus*, *B*. *ladakhensis* and *B*. *longipennis*, had potential suitable and highly suitable habitats towards the Tibetan Plateau of China. However, only one species from this region, *B*. *friseanus*, had a potential distribution towards both Tibet and south-east China. All five southern species, *B*. *bicoloratus*, *B*. *braccatus*, *B*. *breviceps*, *B*. *picipes* and *B*. *trifasciatus*, had potential distributions towards the southern and south-eastern parts of the country. Unlike the other regions, the species from the northern regions had four different types of distribution trends. Two of these species, *B*. *hypocrita* and *B*. *sporadicus*, had potential distributions towards the north-east of China. Four species, *B*. *lantschouensis*, *B*. *patagiatus*, *B*. *longipes* and *B*. *ussurensis*, were present from central to north-east China. Two species, *B*. *ignitus* and *B*. *pyrosoma*, had distributions towards central to northern China. The remaining four species, *B*. *cryptarum*, *B*. *lucorum*, *B*. *muscorum* and *B*. *sibiricus*, had distributions towards north-east and north-west China (see Supplementary Fig. S2).

### Habitat overlap risk analysis

**Figure 4:**
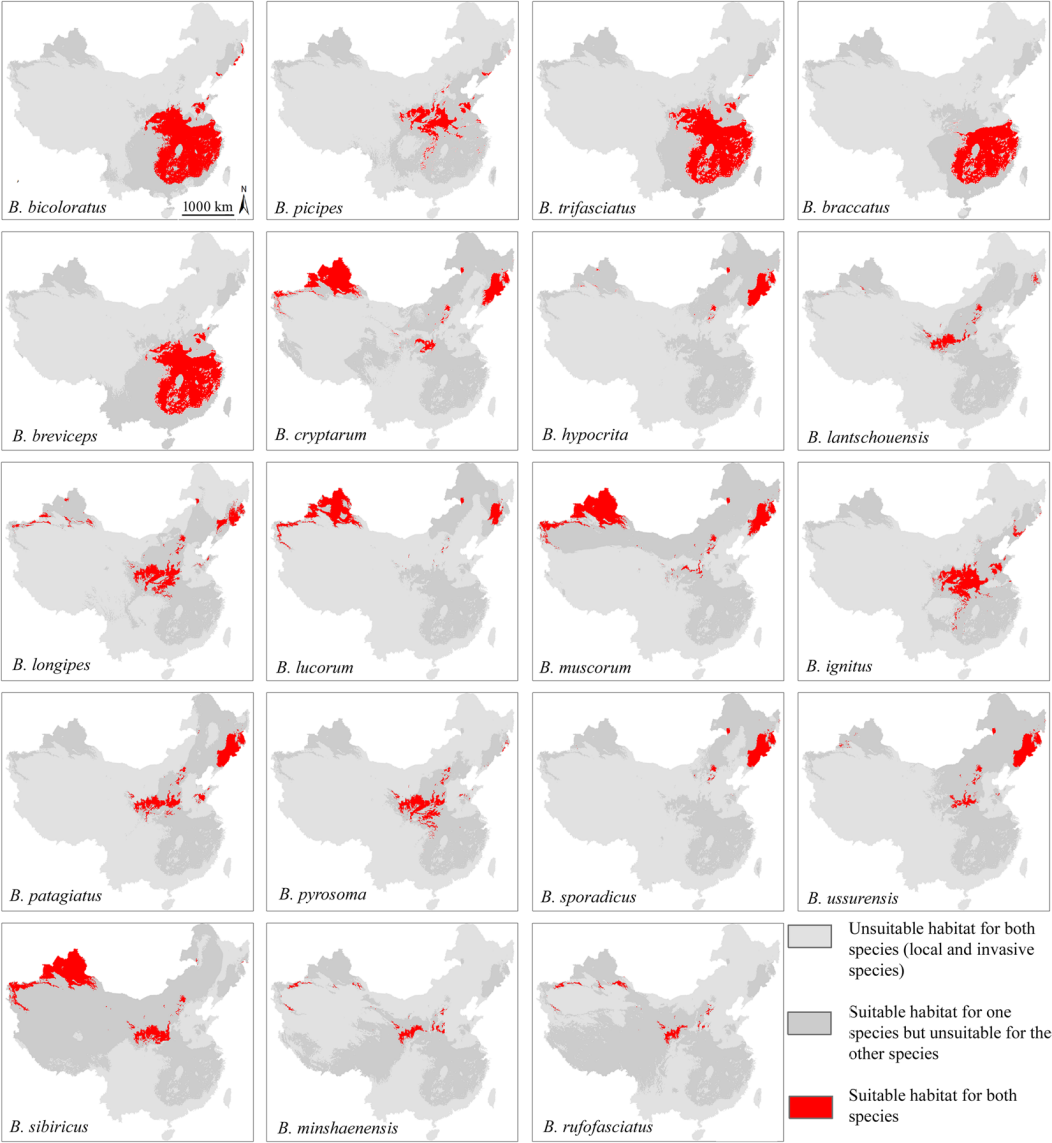
Habitat overlap risk analysis between *Bombus terrestris* and vulnerable native bumblebee species in China. Map was created with ArcGIS v 10.0 (www.arcgis.com).

**Figure 5:**
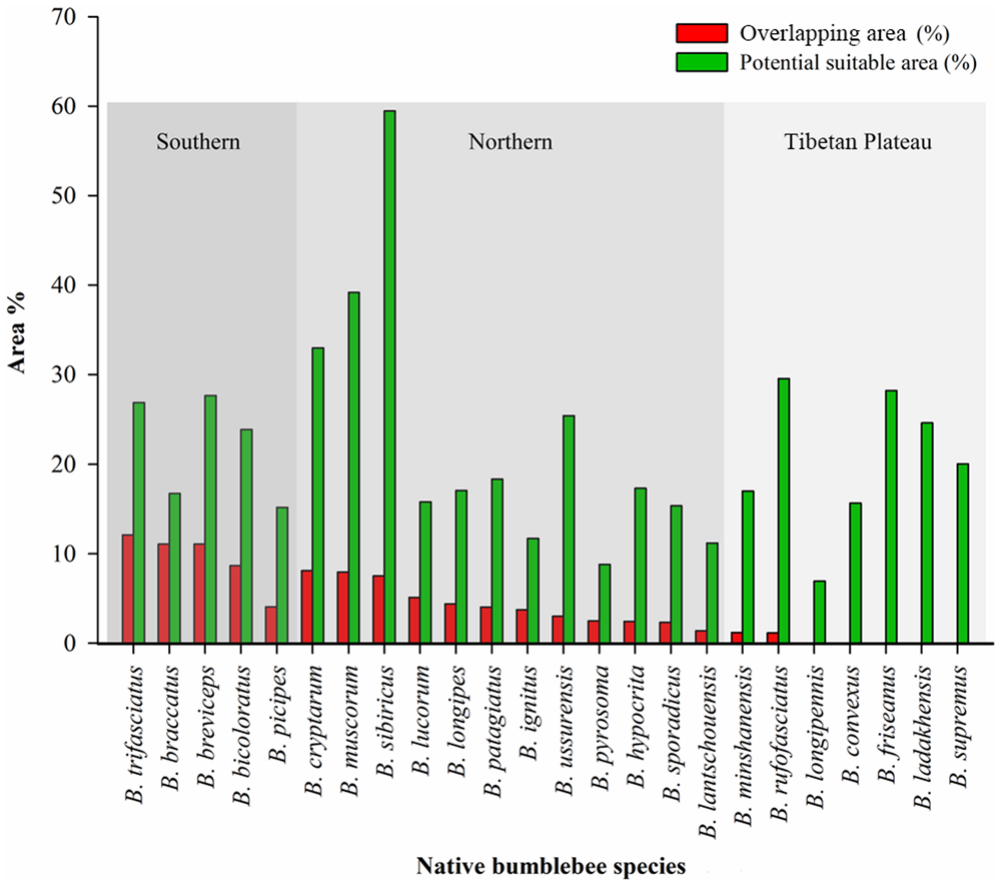
Potential suitable areas vs overlapping habitat areas (%) between native bumblebee species and *B. terrestris*. The three shades of grey representing the “southern”, “northern” and “Tibetan Plateau” regions revealed that the species from the southern region have the most overlapping habitat areas with *B*. *terrestris* followed by the northern region and the Tibetan Plateau.

There were 19 of 24 (79%) native bumblebee species overlapping with *B*. *terrestris* (Table 1). On average, the potential area of overlap between *B*. *terrestris* and native species was 464,900 ± 360,527 km^2^ (mean ± standard deviation). The sensitivity of habitat overlap was found to be greater (89%) for the “closely related” group (biological group) of the same subgenus, *Bombus* s. str., with *B*. *terrestris* compared to the “non-closely related” group (73%) of other subgenera of bumblebee species. For regional species, species from both the “southern” and “northern” regions had maximum overlap (100%) compared to the species from the “Tibetan Plateau” (29%). However, in the overlap areas, the species from the southern region had greater overlap with *B*. *terrestris* than did the species from the northern region, and only two species from the Tibetan Plateau had (slight) overlap with *B*. *terrestris* (Figs 4 and 5).

The spatial distributions of overlapping habitats between native and invasive species were found in all three regions with suitable habitat for *B*. *terrestris*: north-west, central to south-east and north-east within China. All five “southern” species, including *B*. *bicoloratus*, *B. braccatus*, *B. breviceps*, *B*. *picipes* and *B*. *trifasciatus*, mainly had potential overlapping habitats in central to south-east China. Six of the 12 northern species, *B*. *cryptarum*, *B*. *hypocrita*, *B*. *lantschouensis*, *B*. *longipes*, *B*. *lucorum* and *B*. *muscorum*, had potential overlapping habitats in all three regions with habitats suitable for *B*. *terrestris*. Five other “northern” species, *B*. *ignitus*, *B*. *patagiatus*, *B*. *pyrosoma*, *B*. *sporadicus* and *B*. *ussurensis*, had potential overlapping habitats towards central to north-east China. However, only one “northern” species, *B*. *sibiricus*, had a potential overlapping habitat towards central to north-west China. Similarly, two “Tibetan Plateau” species, *B*. *minshaenensis* and *B*. *rufofasciatus*, had potential overlapping habitats towards central to north-west China.

Eight of the nine species from the “closely related” group had overlapping habitats, and the range of overlapping habitat area was 114,425~776,801 km^2^ (1~9%). However, 11 of the 15 species from the “non-closely” related group had overlapping habitats, and the range of overlapping habitat area was 109,520~1,160,506 km^2^ (1~12%). Although all species in the “southern” and “northern” regional groups had overlapping habitats, the species in the “southern” group had a greater area of overlapping habitats, from 389,307^~^1,160,507 km^2^ (4~12%), than that, from 132,155 km^2^~776,801 km^2^ (1~8%), of the species in the “northern” group. However, the species from the “Tibetan Plateau” had an overlapping habitat area that ranged from 109,520~114,425 km^2^, which was only 1% of the whole area of China (Table 1, Fig. 5).

## Discussion

In comparison to a single-model approach, summed distribution maps can provide more accurate estimations of the spatial distributions of species^40^. In the present study, five modelling approaches were implemented here to accurately determine the spatial distributions of *B*. *terrestris* throughout East Asian countries and of 24 local bumblebee species within China. To assess the accuracy of such models, TSS, AUC and kappa statistics values can be used. However, there are inherent limitations to using kappa statistics^41^, and AUC approaches do not allow the prediction of detection differences between more than two models^42^. The prevailing problem in kappa statistics involves their dependency on the ratio between the proportion of correctly predicted presences (sensitivity) and the proportion of correctly predicted absences (specificity)^41^. To avoid these problems, TSS values were calculated to assess model accuracy in the present study. The different ranges of TSS values among the different modelling approaches are due to the different mechanistic features of the models, and typically, these different features lead to model differences in predicted distributions^37,43^. These TSS ranges facilitated our evaluation of the modelling approaches used here. MaxEnt attained the highest performance among the modelling approaches, which is consistent with previous studies^40,44–47^.

In contrast to the findings of Acosta^19^, where East Asian countries including Japan, South Korea, North Korea and south-eastern coastal parts of China were considered suitable for *B*. *terrestris* invasion, our habitat suitability modelling approaches predicted that Mongolia as well as north-west, central to south-east and north-east parts of China (covering 24 provinces) are also suitable for invasion by *B*. *terrestris*. These regions are physiographically similar to Europe and have already been predicted to be climatically suitable for *B*. *terrestris*^48,49^. However, none of our models predicted that habitat susceptibility to invasion would occur only towards the south-eastern area. Our results are supported by the presence of some new records of *B*. *terrestris* in the predicted suitable habitats in China as well as in neighbouring countries surrounding China.

Interspecific mating between “closely related” invasive and native bumblebee species may occur, disturbing reproduction and reducing local bumblebee diversity. Likewise, in Japan, the invasive species *B*. *terrestris* has been found to mate with its closely related local species *B*. *hypocrita* and is responsible for the reduction in the local bumblebee population^50^. In addition, hybrid-mating disturbance has been found between *B*. *terrestris* males and native queens of Chinese *B*. *lantschouensis* under artificial conditions and may also occur in the field^51^. Although eight of the nine species from the “closely related” group (species of the same subgenus, *Bombus* s. str.) within China overlapped with *B*. *terrestris*, four species of the “non-closely related” group from the subtropical region of south China, *B*. *trifasciatus*, *B*. *braccatus*, *B*. *breviceps* and *B*. *bicoloratus*, had greater areas of overlapping habitat than did any of the “closely related” species” (Fig. 5). This habitat overlap between the invasive species and native species from the “non-closely related” group might be due to their ecological similarities^30^. More suitable similar habitats are responsible for the maximum overlap between *B*. *terrestris* and local bumblebees. More importantly, *B*. *muscorum* from the “non-closely related” group is already categorised as vulnerable in Britain^52^ and is vulnerable in China. Given that the foraging distance of *B*. *muscorum* (which forages mostly within 500 m of its nest) is shorter than that of *B*. *terrestris* (which forages mostly over 2 km from its nest)^53^, Habitat overlap with *B*. *terrestris* may be harmful for this local species because *B*. *terrestris* may be able to outcompete *B*. *muscorum* for food resources within areas close to the latter’s nests.

Alarmingly, eight species endemic to China^23^, *B*. *braccatus*, *B*. *convexus*, *B*. *longipes*, *B*. *minshaenensis*, *B*. *picipes*, *B*. *pyrosoma*, *B*. *supremus* and *B*. *trifasciatus*, are threatened by habitat overlap with the invasive *B*. *terrestris*. These species are important pollinators of many wild flowers and crops within China^24^, and endemic species are considered more important to local biodiversity than are non-native species^54^. Therefore, a management strategy should be implemented that prioritises the conservation of these endemic bumblebees.

Similarly, some of the overlapping species, specifically, *B*. *braccatus*, *B*. *cryptarum*, *B*. *hypocrita*, *B*. *lucorum* and *B*. *muscorum*, were “rare” in terms of abundance in this study (Table 1). Two of them, *B*. *lucorum* and *B*. *muscorum*, are also “moderately rare” in Hungry and Russia^55–58^. These observations highlight the potential for declines in vulnerable species within a country. However, due to species differences in food selection, the conservation strategies used for other species will not be effective for these rare species. Thus, from a conservation point of view, specific conservation planning is urgently needed to conserve these bumblebee species^59,60^.

**Figure 6:**
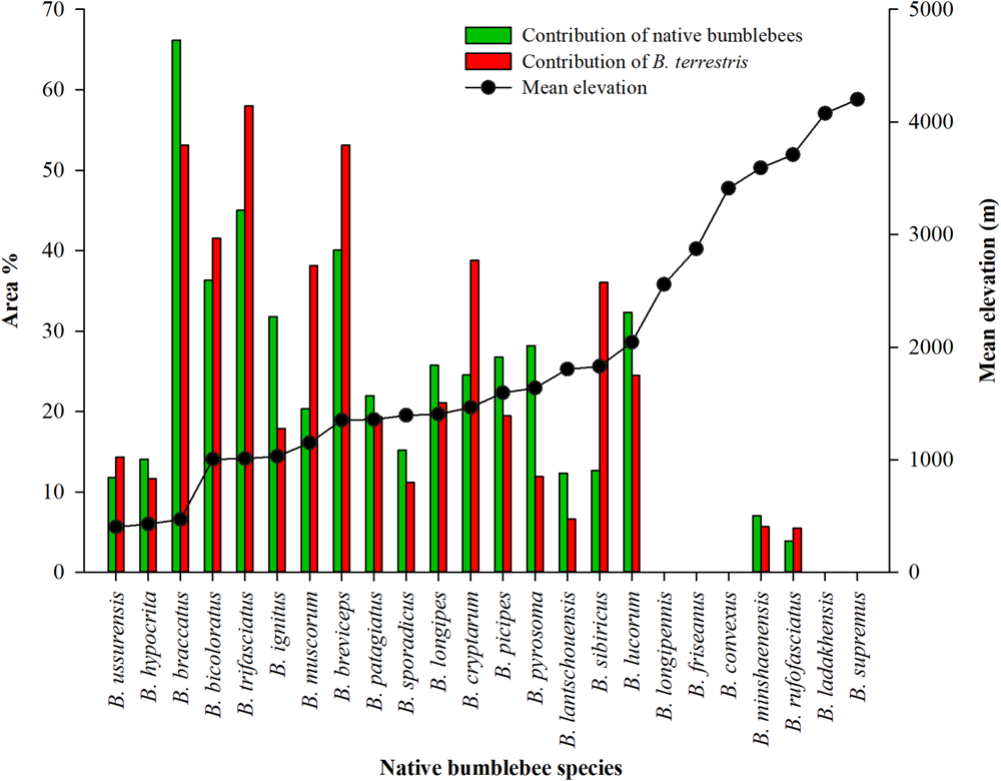
The proportion of contribution of the potential suitable habitat areas (%) of both *B*. *terrestris* and native bumblebee species that overlap at different mean elevations.

The native bumblebee species from the “southern”, “northern” and “Tibetan Plateau” regions were distributed at different altitudes (Fig. 6). All the native species from the southern and northern regions at mean elevations <2500 metres had overlapping habitats. However, for the high, cold species from the Tibetan Plateau at mean elevations >2500, only two species, *B*. *minshaenensis* and *B*. *rufofasciatus*, had a very small proportion of overlapping habitats (<1% areas) with *B*. *terrestris*, and the remaining species were not overlapping (Fig. 6). This scenario might indicate that the high, cold region of the Tibetan Plateau has the least amount of suitable habitat for the invasive species or that these species have ecological dissimilarities^30^.

Our current findings regarding the distribution of *B*. *terrestris* and its overlapping habitats with different groups of local bumblebees showed that the local bumblebees of other East Asian countries might also be threatened by habitat overlap and loss of native biodiversity. Proper management strategies and coordinated implementation of regulations related to the introduction of *B*. *terrestris*^61^ should be considered for all East Asian countries to conserve the natural biodiversity of local bumblebee species within this region.

## Methods

### Modelled species

**Figure 7:**
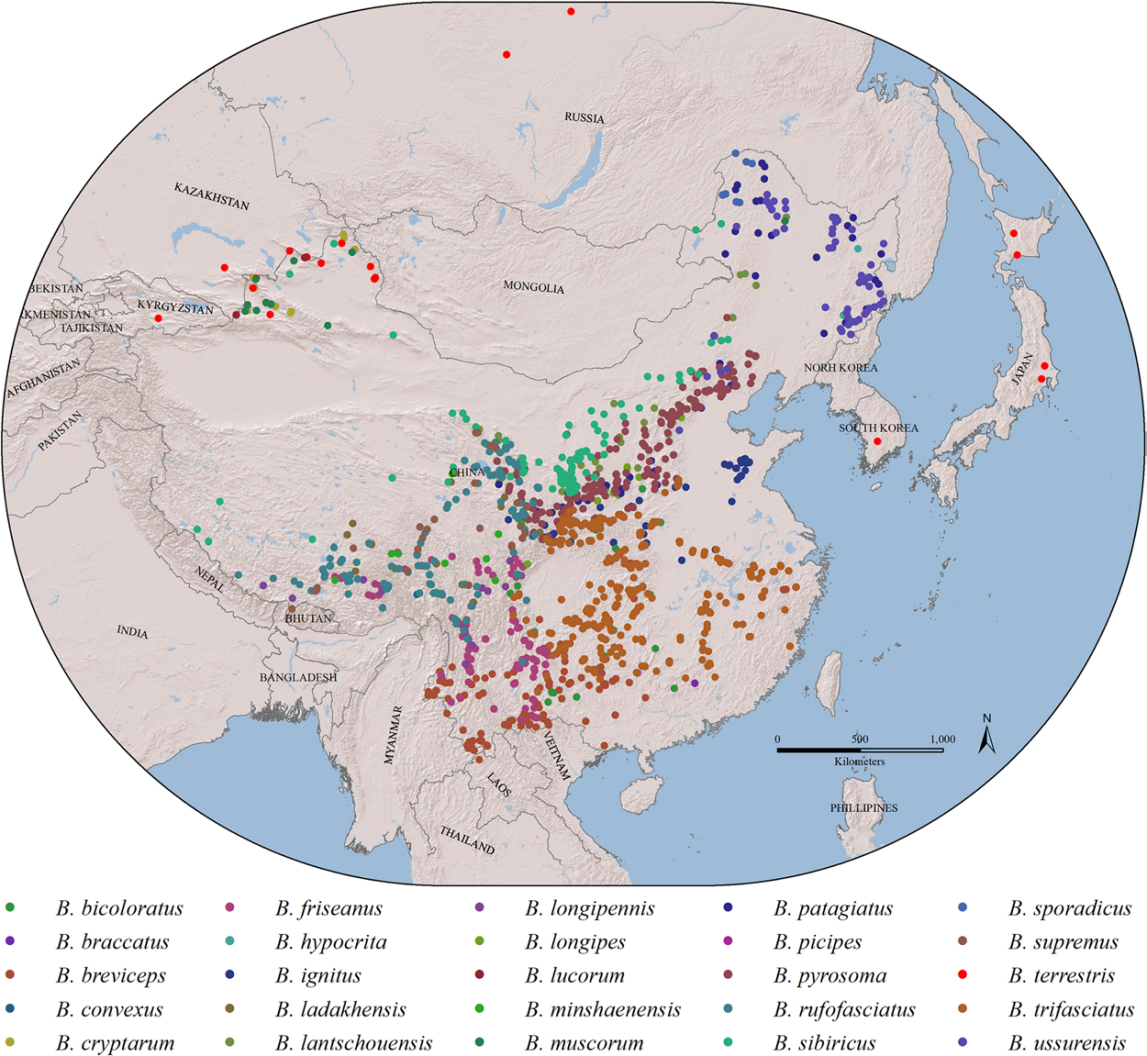
Relief map of the Chinese mainland showing the sample sites of the 25 bumblebee species with the different colourful spots representing the different species in this study. The records of *B. terrestris* for areas surrounding China are from (www.discoverlife.org). The international boundaries are shown in grey with the names of countries in capital letters. The map was created with ArcGIS v 10.0 (www.arcgis.com).

Records of *B*. *terrestris* were based on its natural distribution within China and surrounding countries, such as Kazakhstan, Kyrgyzstan and Russia, as well as within the areas close to China such as Japan and South Korea, where it is invasive (www.discoverlife.org) (Fig. 7). For the local bumblebees in China, we selected 24 native bumblebee species and *B*. *terrestris* with a total of 26,497 specimen records from the IAR (CAAS Institute of Apiculture Research) collection that covers the whole region of the Chinese mainland (Table 1). These species were represented in >50% of the records of the 125 bumblebee species in China and were selected by two principles, 1) representatives of two groups: species “closely related” to *B*. *terrestris*, i.e., from the subgenus *Bombus* s. str., all known in China (n = 10) and “non-closely related” species, i.e., species of other bumblebee subgenera (n = 14), and 2) representatives of the three major regions of China, i.e., the high cold “Tibetan Plateau” and the temperate “northern” and subtropical “southern” regions (Table 1).

### Environmental layers and modelling procedures

In our modelling, we used 19 bioclimatic and elevation layers obtained from www.worldclim.org with a grid cell size of 5 arc-min resolution (≈10 km^2^) of the WGS1984 projection^62^ with full coverage of East Asian countries. We converted all these layers into ASCII format to use in our modelling software with ArcGIS *v* 10.0. We calculated Pearson’s correlation coefficients with the species distribution modelling toolbox^63^ of ArcGIS *v* 10.0 to reduce the collinearity among these bioclimatic layers, and we retained only seven bioclimatic variables together with the elevation layers, which all yielded Pearson correlation coefficient (r) values < 0.8, to avoid overfitting the model (see Supplementary Table S2). Similarly, we used a spatial rarefy tool of the SDM toolbox^63^ to remove all the presence points of species that have distances <10 km^2^ to avoid spatial autocorrelation among these occurrence records of bumblebee species during modelling (Table 1). All the occurrence records were converted into comma separated value (CSV) comma delimited and text tab delimited formats for the use in MaxEnt *v* 3.3.3k and OpenModeller Desktop software *v* 1.1.0, respectively. For MaxEnt software, we prepared a biased file of all 25 species occurrence records for background point selection using ArcGIS *v* 10.0.

Five modelling algorithms, MaxEnt^64^, Envelope Score^65^, Environmental Distance^66^, GARP^67^ and SVM^68^, were implemented to increase the reliability of the potential distributions as well as to identify areas of overlap between the 24 native bumblebee species and *B*. *terrestris*^40^. These models were selected mainly for their different mechanistic features^69^. Due to the variation in the distribution data of a species, there is error in the estimation of the spatial distribution of species by different modelling approaches. As a result, similar data used in different modelling approaches can yield very different results^70^. Therefore, testing more than one model is preferable for addressing the error^38^. All modelling approaches have inherent differences in the generation of distribution predictions^37,43^. By comparing their results, one can retain only those modelling approaches that yielded similar and accurate species distributions^37^. Among the algorithms considered in our study, the envelope score and environmental distance algorithms are simpler algorithms that predict species distributions only on the basis of presence data. In contrast, the remaining three modelling approaches, MaxEnt, SVM and GARP, are more complex and involve artificial intelligence methods. These approaches have been shown to reliably predict species occurrence^43^. Two software programs, MaxEnt *v* 3.3.3k and OpenModeller Desktop *v* 1.1.0, were used to model the distributions of bumblebee species^71^. We developed our models using 75% of our data for training and the remaining 25% for testing our models.

In contrast to the Pearson^72^ approach, where the lowest presence threshold (LPT) is used, in this study, only receiver operating characteristics curve (ROC) threshold values were used to cut the modelled suitability matrices. This approach determines the balance between omission and commission errors while determining the spatial distributional ranges of a species^40^. Furthermore, TSS values were used (which range from −1 to 1) to evaluate the accuracies of the five modelling approaches^26,41^. Here, values close to zero or negative mean that the distribution is not much better than random, whereas values close to one indicate almost perfect agreement between the modelling prediction and distribution. Normally, TSS values ≥0.5 are considered acceptable^40^. Finally, we selected only the distribution modelling outputs that yielded TSS values ≥0.5 (see Supplementary Table S1) to develop the “summed” distribution maps within ArcGIS *v* 10.0 that produced relatively realistic and potentially accurate spatial distributions of the species. In this study, 10,000 random pseudo-absences were used. We categorised our final “summed” potential distribution maps into three categories, 1 = unsuitable habitats, 2 = suitable habitats and 3 = high suitable habitats, based on their specific optimum threshold cut off values within ArcGIS *v* 10.0. (Table 1).

### Assessment of overlapping habitats

To assess the habitat overlap of both invasive and local bumblebee species, the Youden Index was applied, calculated with following the formula: sensitivity + specificity −1^39,73^. This index is used to determine the cut-off point for the models. A high Youden Index value indicates good performance of the model^39^. The final “summed” potential distribution maps were divided into suitable (>threshold value of Youden Index) and unsuitable (<threshold value of Youden Index) categories within ArcGIS *v* 10.0. The threshold value for each species is calculated as the average TSS values of all those modelling algorithms with TSS values >0.5 (see Supplementary Table S1). Then, the distribution maps of the 24 local species were overlaid with the final “summed” distribution map of *B*. *terrestris*, and the cells were reclassified into three categories using the Arc toolbox of ArcGIS *v* 10.0: 1 = unsuitable habitat for both species (local and invasive species), 2 = suitable habitat for one species but unsuitable for the other species, and 3 = suitable habitat for both species (Fig. 4). The optimum cut off values for all the 25 bumblebee species are shown in Table 1. All the grid cells that have values below this average threshold are considered unsuitable for species presence or vice versa.

## Electronic supplementary material

Supplementary Information
