# Supplementary material for: Habitat suitability for the invasion of Bombus terrestris in East Asian countries: A case study of spatial overlap with local Chinese bumblebees
Source: Sci Rep. 2018 Jul 23;8:11035. doi: 10.1038/s41598-018-29414-6 (PMC6056460; doi:10.1038/s41598-018-29414-6)
Supplement: Supplementary file 1 — Supplementary Information [file 41598_2018_29414_MOESM1_ESM.pdf]

Habitat suitability for the invasion of *Bombus terrestris* in East Asian countries: A case study of spatial overlap with local Chinese bumblebees

Muhammad Naeem, Xiaolong Yuan, Jiaxing Huang*, Jiandong An*

*anjiandong@caas.cn (JA); huangjiaxing@caas.cn (JH)

Key Laboratory for Insect-Pollinator Biology of the Ministry of Agriculture, Institute of Apicultural Research, Chinese Academy of Agricultural Sciences, Beijing 100093, China.

Supplementary Table S1 True skilled statistics (TSS) values of five modelling algorithm approaches, only those models for each species (highlighted in bold) were selected to develop the summed distribution maps that have TSS values > 0.5

Species	Envelope Score	Environmental distance	GARP	MaxEnt	SVM
B. bicoloratus	0.27	**0.59**	**0.66**	**0.78**	0.01
B. braccatus	**0.78**	**0.87**	**0.61**	**0.91**	0
B. breviceps	0.26	**0.65**	**0.72**	**0.81**	0.48
B. convexus	**0.54**	**0.76**	**0.82**	**0.85**	0
B. cryptarum	0.24	**0.91**	**0.86**	**0.55**	0.02
B. friseanus	0.19	**0.83**	**0.79**	**0.80**	0.42
B. hypocrita	**0.82**	**0.96**	**0.93**	**0.93**	0
B. ignitus	0.03	0.39	0.48	**0.72**	0.12
B. ladakhensis	0	**0.92**	**0.72**	**0.62**	0
B. lantschouensis	0.24	**0.55**	**0.64**	**0.64**	0.01
B. longipennis	0.09	0.42	0.35	**0.71**	0.00
B. longipes	0.11	**0.55**	**0.59**	**0.87**	0.01
B. lucorum	0.43	**0.97**	0.36	0.40	0.00
B. minshaenensis	**0.53**	**0.79**	**0.76**	**0.84**	0.02
B. muscorum	0.35	**0.96**	**0.75**	**0.67**	0
B. patagiatus	0.19	0.48	0.48	**0.71**	0
B. picipes	0.01	0.27	0.35	**0.71**	0
B. pyrosoma	0.02	0.43	0.47	**0.74**	0

B. rufofasciatus	0.12	**0.75**	**0.74**	**0.67**	0.03
B. sibiricus	0.11	**0.63**	**0.70**	**0.73**	0.05
B. sporadicus	**0.86**	**0.95**	**0.95**	**0.85**	0.00
B. supremus	**0.58**	**0.83**	**0.82**	**0.73**	0.00
B. terrestris	**0.77**	**0.79**	**0.58**	**0.87**	0.17
B. trifasciatus	0.19	**0.64**	**0.69**	**0.79**	0.38
B. ussurensis	0.38	**0.86**	**0.93**	**0.85**	**0.98**

Supplementary Table S2 Bioclimatic variables used for developing the spatial distribution modelling in East Asian countries.

#	**Variables used for the modeling**
1	annual mean temperature
2	mean diurnal range [mean of monthly (max temp - min temp)]
3	isothermality (bio2/bio7) (* 100)
4	temperature seasonality (standard deviation *100)
5	precipitation of wettest month
6	precipitation of driest month
7	precipitation seasonality (coefficient of variation)
8	elevation

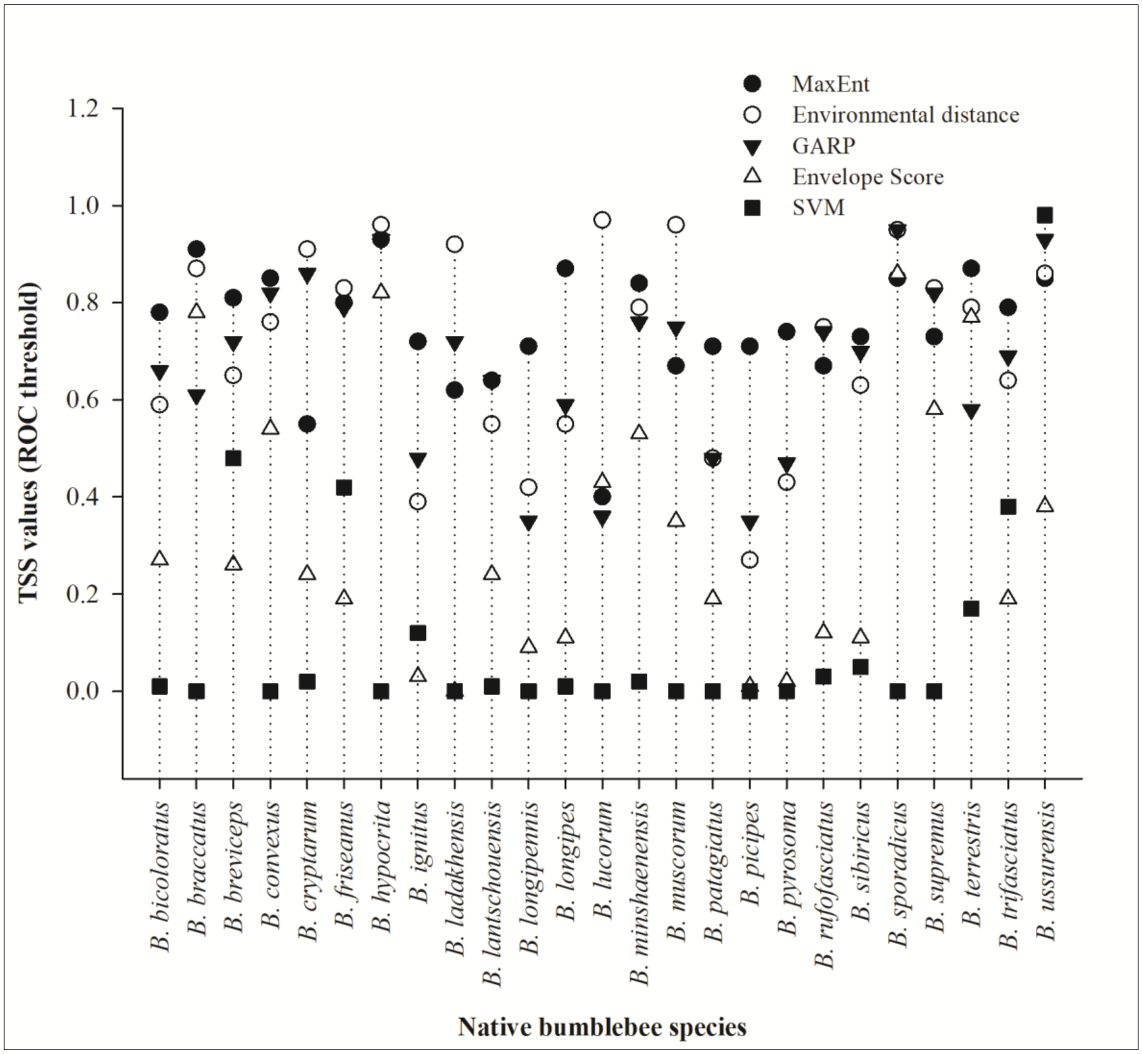

Supplementary Figure S1. True skilled statistics (TSS) values of the five modelling algorithms against the 25 bumblebee species

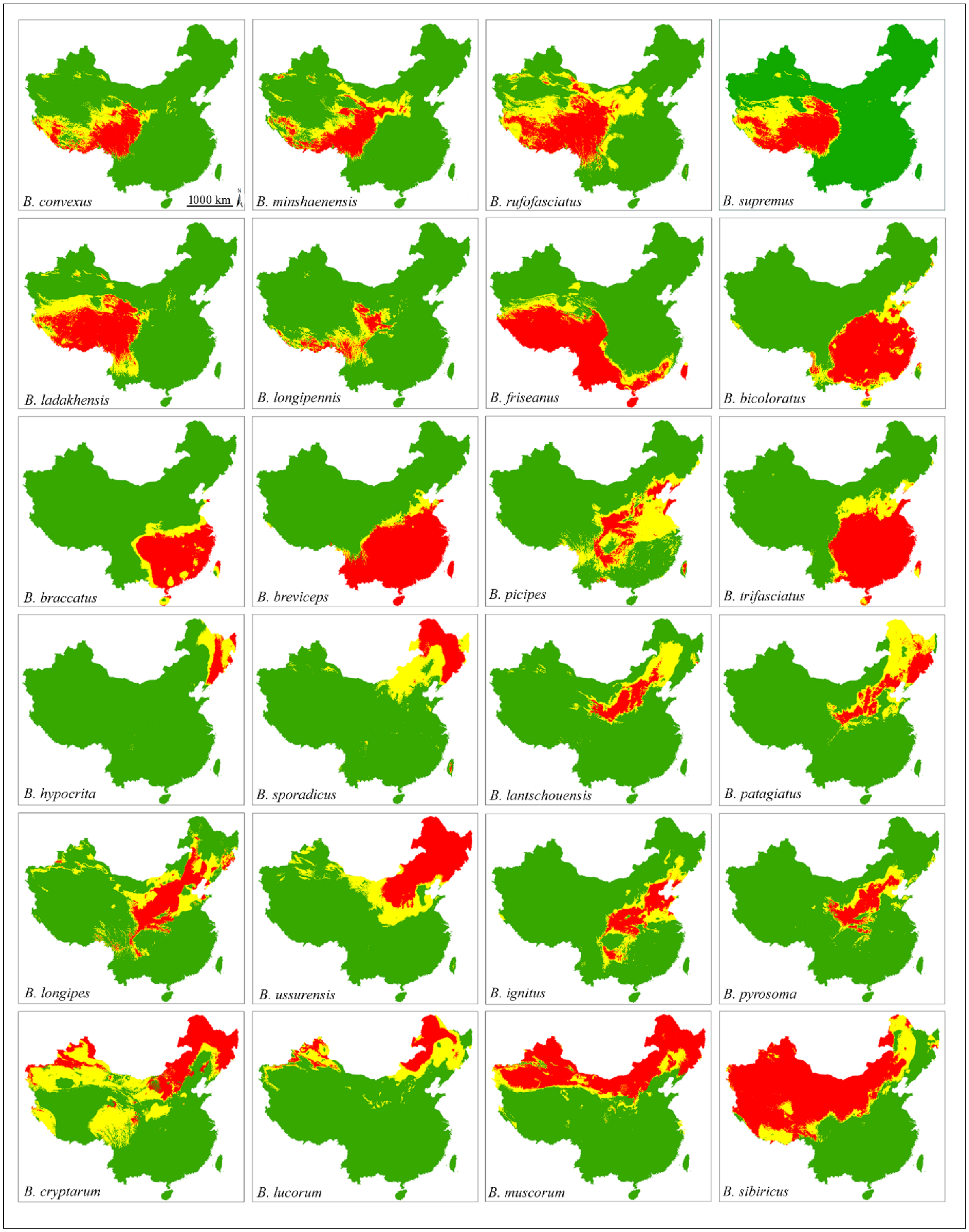

Supplementary Figure S2. Potential distribution modelling of the 24 native bumblebee species within China in this study. Red represents the highly suitable habitat, yellow represents suitable habitat, and green represents unsuitable habitat. The map was created with ArcGIS *v* 10.0 (www.arcgis.com).
